# (*R*)-*N*-Methyl-4-[2-(methyl­sulfan­yl)pyrimidin-4-yl]-1-(tetra­hydro­furan-3-yl)-1*H*-pyrazol-5-amine

**DOI:** 10.1107/S1600536809006369

**Published:** 2009-02-28

**Authors:** Zhengyu Liu, Kevin K.-C. Liu, Jeff Elleraas, Arnold L. Rheingold, Antonio DiPasquale, Alex Yanovsky

**Affiliations:** a Pfizer Global Research and Development, La Jolla Labs, 10614 Science Center Drive, San Diego, CA 92121, USA; b Department of Chemistry and Biochemistry, University of California, San Diego, 9500 Gilman Drive, La Jolla, CA 92093, USA

## Abstract

The chiral center at the substituted atom of the tetra­hydro­furanyl ring in the title compound, C_13_H_17_N_5_OS, has an *R* configuration. The methyl­sulfanylpyrimidine group and the pyrazole ring are almost coplanar [the maximum deviation from this plane is 0.070 (4) Å], the N—Me substituent being displaced from the methyl­sulfanylpyrimidine-pyrazole plane by 0.880 (4) Å. The secondary amine group participates in an intra­molecular hydrogen bond with the pyrimidine N atom in position 3.

## Related literature

For the structures of related pyrimidine derivatives with similar intra­molecular hydrogen bonds, see: Golic *et al.* (1993 ▶).
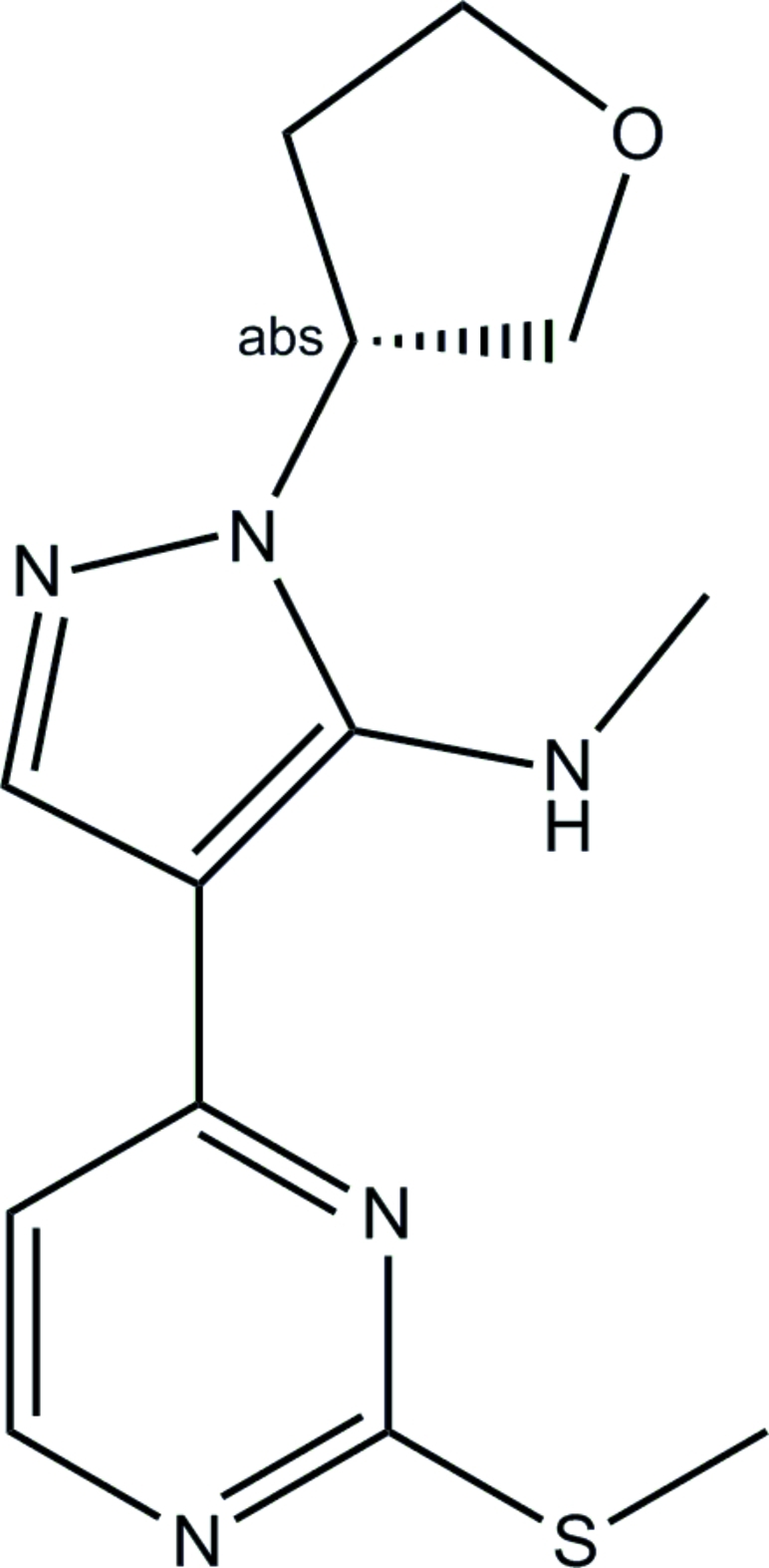


## Experimental

### 

#### Crystal data

C_13_H_17_N_5_OS
*M* *_r_* = 291.38
Orthorhombic, 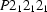

*a* = 6.6209 (4) Å
*b* = 14.0757 (9) Å
*c* = 14.6793 (10) Å
*V* = 1368.02 (15) Å^3^
*Z* = 4
Cu *K*α radiation
μ = 2.14 mm^−1^
*T* = 100 K
0.12 × 0.10 × 0.06 mm

#### Data collection

Bruker Kappa APEXII CCD area-detector diffractometer
Absorption correction: multi-scan (*SADABS*; Bruker, 2001 ▶) *T*_min_ = 0.783, *T*_max_ = 0.882
9765 measured reflections
2419 independent reflections
2388 reflections with *I* > 2σ(*I*)
*R*_int_ = 0.021

#### Refinement

*R*[*F* ^2^ > 2σ(*F* ^2^)] = 0.021
*wR*(*F* ^2^) = 0.057
*S* = 1.06
2419 reflections
187 parameters
H atoms treated by a mixture of independent and constrained refinement
Δρ_max_ = 0.22 e Å^−3^
Δρ_min_ = −0.16 e Å^−3^
Absolute structure: Flack (1983 ▶), 964 Friedel pairs
Flack parameter: 0.061 (12)

### 

Data collection: *APEX2* (Bruker–Nonius, 2004 ▶); cell refinement: *SAINT* (Bruker–Nonius, 2004 ▶); data reduction: *SAINT*; program(s) used to solve structure: *SIR2004* (Burla *et al*., 2005 ▶); program(s) used to refine structure: *SHELXL97* (Sheldrick, 2008 ▶); molecular graphics: *ORTEP-32* (Farrugia, 1997 ▶); software used to prepare material for publication: *WinGX* (Farrugia, 1999 ▶).

## Supplementary Material

Crystal structure: contains datablocks global, I. DOI: 10.1107/S1600536809006369/tk2375sup1.cif

Structure factors: contains datablocks I. DOI: 10.1107/S1600536809006369/tk2375Isup2.hkl

Additional supplementary materials:  crystallographic information; 3D view; checkCIF report

## Figures and Tables

**Table 1:** Hydrogen-bond geometry (Å, °)

*D*—H⋯*A*	*D*—H	H⋯*A*	*D*⋯*A*	*D*—H⋯*A*
N3—H3*N*⋯N5	0.858 (15)	2.157 (15)	2.8510 (14)	137.9 (14)

**Fig. 1.**
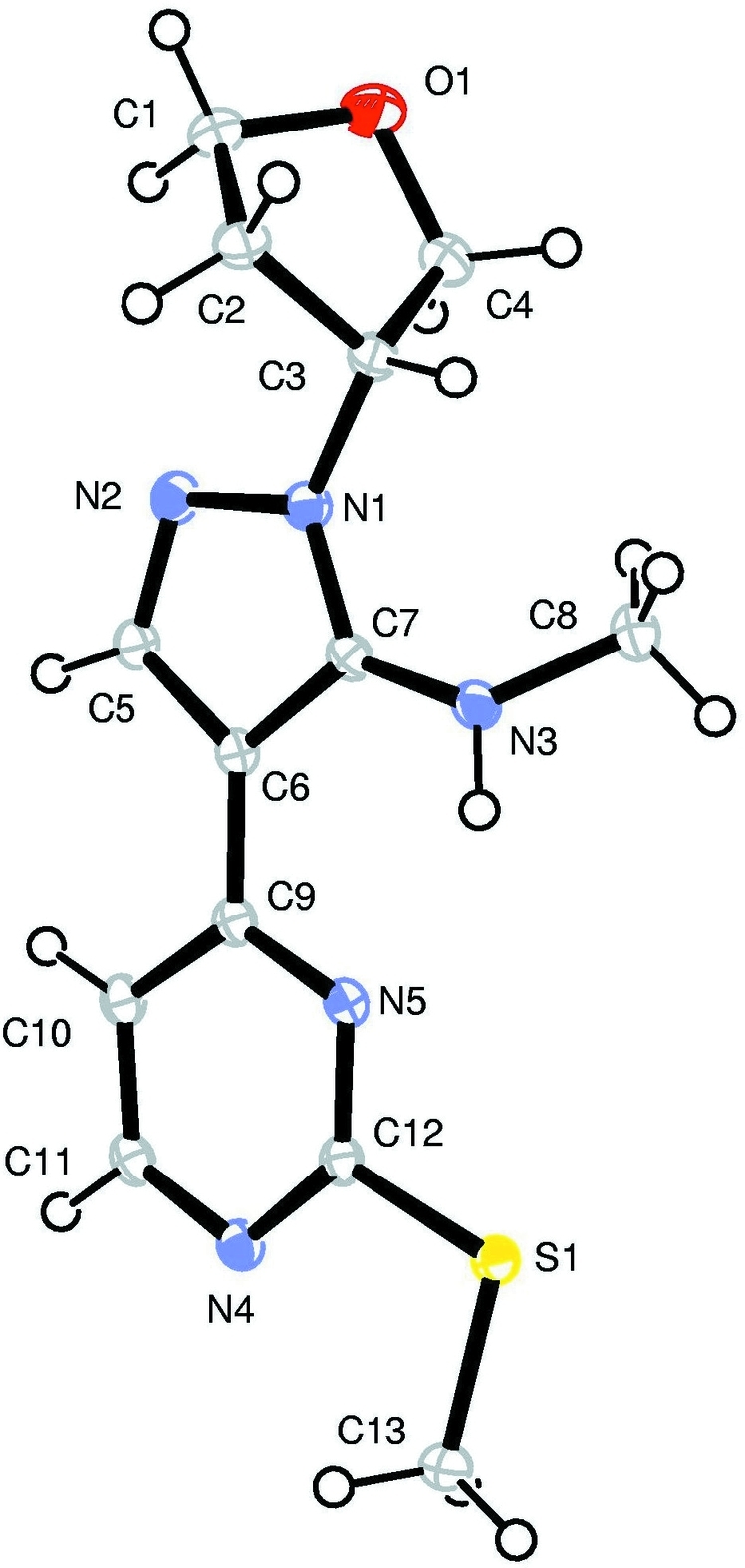
Molecular structure of (I), showing 50% probability displacement ellipsoids and atom numbering scheme. H atoms are drawn as circles with arbitrary small radius.

## supplementary crystallographic information

### Comment

The title compound (I) was obtained by *N*-methylation of 4-(2-(methylsulfanyl)pyrimidin-4-yl)-1-(tetrahydrofuran-2-yl)-1*H*-pyrazol-5-amine with methyl iodide. The racemic product was then separated with the help of chiral chromatography; (I) was collected as the earlier fraction when eluted with isopropyl alcohol using the Chiralpak column (99% ee; [α]_D_^20^ = -191.4°)

The present X-ray study unambiguously established the *R*-configuration of the chiral center at the C3 atom (Fig. 1).

The methylsulfanylpyrimidine group and pyrazolyl ring lie approximately in one plane. The maximum deviation from this plane being 0.070 (4) Å for the C13 atom; the displacement of methyl-C8 atom from this plane is 0.880 (4) Å. The orientation of the tetrahydrofuran ring can be characterized by the dihedral angle of 98.1 (3)° formed by the pyrimidine-pyrazolyl plane with the C2—C3—C4 plane.

The secondary amino group forms an intramolecular hydrogen bond with the N5 atom of the pyrimidine ring, Table 1, the geometry of this bond is similar to that observed in ethyl (*Z*)-2-amino-3-(4-pyrimidinyl)propenoate (Golic *et al.*, 1993).

### Experimental

A solution of -(2-(methylsulfanyl)pyrimidin-4-yl)-1-(tetrahydrofuran-2-yl)-1*H*-pyrazol-5-amine (8.32 g, 30.0 mmol) in anhydrous THF (80 ml) was added dropwise to a suspension of hexane-washed NaH (60% dispersion in mineral oil, 1.92 g, 48.0 mmol) in anhydrous THF (20 ml) at room temperature. The resulting orange reaction mixture was stirred under nitrogen for 30 minutes; thereafter MeI (5.96 g, 42.0 mmol) was added dropwise. The reaction mixture was stirred at room temperature under nitrogen overnight and then quenched with aqueous NH_4_Cl (100 ml). EtOAc (200 ml) was added and layers were separated. The organic extract was washed with brine, dried over sodium sulfate, and concentrated to give the crude product, which was purified by flash chromatography using 20–50% EtOAc in hexane to afford 5.85 g (67%) of yellow solid. The racemic product thus obtained was subjected to chiral chromatography on Chiralpak AS—H 21.2 *x* 250 mm column with 15% IPA in CO_2_ at 140 bar as eluent (temp = 35°C; flow = 60 ml/min; UV detection at 260 nm). Two fractions corresponding to each of the enantiomers (Peak1 and Peak2) were collected and evaporated to dryness; specific rotation [α]_D_^20^ was measured in methanol solution and yielded the values of -191.4° and +224.1°, respectively. The enantiomer collected as Peak 1 was recrystallized from EtOAc/hexane to yield single crystals.

### Refinement

All H atoms bonded to C atoms were placed in geometrically calculated positions (C—H 0.95 Å, 0.98 Å, 0.99 Å, and 1.00 Å for aromatic-, methyl-, methylene- and methyine-H atoms, respectively) and included in the refinement in the riding model approximation. The H3N atom was located in a difference Fourier map and refined isotropically [N3—H3N 0.858 (15) Å]. The *U*_iso_(H) were set to 1.2*U*_eq_ of the carrying atom for non-methyl and amine, and 1.5*U*_eq_ for methyl-H atoms.

### Crystal data

**Table:**

C_13_H_17_N_5_OS	*F*(000) = 616
*M**_r_* = 291.38	*D*_x_ = 1.415 Mg m^−^^3^
Orthorhombic, *P*2_1_2_1_2_1_	Cu *K*α radiation, λ = 1.54178 Å
Hall symbol: P 2ac 2ab	Cell parameters from 8684 reflections
*a* = 6.6209 (4) Å	θ = 3.1–68.1°
*b* = 14.0757 (9) Å	µ = 2.14 mm^−^^1^
*c* = 14.6793 (10) Å	*T* = 100 K
*V* = 1368.02 (15) Å^3^	Block, colorless
*Z* = 4	0.12 × 0.10 × 0.06 mm

### Data collection

**Table:**

Bruker Kappa APEXII CCD area-detector diffractometer	2419 independent reflections
Radiation source: fine-focus sealed tube	2388 reflections with *I* > 2σ(*I*)
graphite	*R*_int_ = 0.021
φ and ω scans	θ_max_ = 67.9°, θ_min_ = 4.3°
Absorption correction: multi-scan (*SADABS*; Bruker, 2001)	*h* = −7→7
*T*_min_ = 0.783, *T*_max_ = 0.882	*k* = −16→16
9765 measured reflections	*l* = −11→17

### Refinement

**Table:**

Refinement on *F*^2^	Hydrogen site location: inferred from neighbouring sites
Least-squares matrix: full	H atoms treated by a mixture of independent and constrained refinement
*R*[*F*^2^ > 2σ(*F*^2^)] = 0.021	*w* = 1/[σ^2^(*F*_o_^2^) + (0.0319*P*)^2^ + 0.2281*P*] where *P* = (*F*_o_^2^ + 2*F*_c_^2^)/3
*wR*(*F*^2^) = 0.057	(Δ/σ)_max_ = 0.001
*S* = 1.06	Δρ_max_ = 0.22 e Å^−^^3^
2419 reflections	Δρ_min_ = −0.16 e Å^−^^3^
187 parameters	Extinction correction: *SHELXL97* (Sheldrick, 2008), Fc^*^=kFc[1+0.001xFc^2^λ^3^/sin(2θ)]^-1/4^
0 restraints	Extinction coefficient: 0.0024 (2)
Primary atom site location: structure-invariant direct methods	Absolute structure: Flack (1983), 964 Friedel pairs
Secondary atom site location: difference Fourier map	Flack parameter: 0.061 (12)

### Special details

**Table:**

Geometry. All e.s.d.'s (except the e.s.d. in the dihedral angle between two l.s. planes) are estimated using the full covariance matrix. The cell e.s.d.'s are taken into account individually in the estimation of e.s.d.'s in distances, angles and torsion angles; correlations between e.s.d.'s in cell parameters are only used when they are defined by crystal symmetry. An approximate (isotropic) treatment of cell e.s.d.'s is used for estimating e.s.d.'s involving l.s. planes.
Refinement. Refinement of *F*^2^ against ALL reflections. The weighted *R*-factor *wR* and goodness of fit *S* are based on *F*^2^, conventional *R*-factors *R* are based on *F*, with *F* set to zero for negative *F*^2^. The threshold expression of *F*^2^ > σ(*F*^2^) is used only for calculating *R*-factors(gt) *etc*. and is not relevant to the choice of reflections for refinement. *R*-factors based on *F*^2^ are statistically about twice as large as those based on *F*, and *R*- factors based on ALL data will be even larger.

### Fractional atomic coordinates and isotropic or equivalent isotropic displacement parameters (Å^2^)

**Table:**

	*x*	*y*	*z*	*U*_iso_*/*U*_eq_	
C1	0.4029 (2)	0.30990 (12)	0.04871 (10)	0.0272 (3)	
H1A	0.4395	0.2446	0.0676	0.033*	
H1B	0.3269	0.3069	−0.0093	0.033*	
C2	0.2806 (2)	0.35928 (10)	0.12196 (10)	0.0213 (3)	
H2A	0.1860	0.3147	0.1519	0.026*	
H2B	0.2034	0.4134	0.0966	0.026*	
C3	0.4445 (2)	0.39343 (9)	0.18853 (8)	0.0167 (3)	
H3	0.4133	0.4595	0.2093	0.020*	
C4	0.6387 (2)	0.39367 (10)	0.12981 (10)	0.0214 (3)	
H4A	0.7017	0.4575	0.1302	0.026*	
H4B	0.7375	0.3472	0.1539	0.026*	
C5	0.4646 (2)	0.19552 (9)	0.33361 (8)	0.0149 (3)	
H5	0.4649	0.1289	0.3441	0.018*	
C6	0.4610 (2)	0.26374 (9)	0.40411 (9)	0.0138 (3)	
C7	0.4621 (2)	0.35123 (9)	0.35763 (8)	0.0135 (2)	
C8	0.5746 (2)	0.52007 (10)	0.36114 (10)	0.0215 (3)	
H8A	0.6704	0.4970	0.3153	0.032*	
H8B	0.6490	0.5481	0.4122	0.032*	
H8C	0.4865	0.5682	0.3338	0.032*	
C9	0.4629 (2)	0.24930 (9)	0.50139 (9)	0.0135 (3)	
C10	0.4728 (2)	0.15830 (9)	0.54074 (9)	0.0162 (3)	
H10	0.4773	0.1026	0.5042	0.019*	
C11	0.4755 (2)	0.15340 (9)	0.63402 (9)	0.0159 (3)	
H11	0.4826	0.0924	0.6615	0.019*	
C12	0.4595 (2)	0.31224 (9)	0.64505 (8)	0.0136 (2)	
C13	0.4376 (2)	0.37321 (10)	0.82303 (9)	0.0210 (3)	
H13A	0.3289	0.3262	0.8275	0.032*	
H13B	0.4109	0.4256	0.8654	0.032*	
H13C	0.5666	0.3432	0.8386	0.032*	
N1	0.46330 (18)	0.33043 (7)	0.26754 (7)	0.0148 (2)	
N2	0.46738 (18)	0.23371 (7)	0.25178 (8)	0.0166 (2)	
N3	0.4520 (2)	0.44067 (7)	0.39416 (7)	0.0171 (2)	
N4	0.46873 (18)	0.22994 (7)	0.68912 (7)	0.0153 (2)	
N5	0.45642 (17)	0.32731 (7)	0.55510 (7)	0.0142 (2)	
O1	0.57971 (18)	0.36833 (9)	0.03959 (7)	0.0318 (3)	
S1	0.44904 (5)	0.41854 (2)	0.70856 (2)	0.01652 (9)	
H3N	0.456 (3)	0.4369 (11)	0.4524 (10)	0.020*	

### Atomic displacement parameters (Å^2^)

**Table:**

	*U*^11^	*U*^22^	*U*^33^	*U*^12^	*U*^13^	*U*^23^
C1	0.0362 (9)	0.0310 (8)	0.0143 (7)	−0.0093 (7)	−0.0007 (6)	−0.0027 (6)
C2	0.0218 (7)	0.0245 (7)	0.0178 (7)	−0.0019 (6)	−0.0048 (6)	0.0038 (6)
C3	0.0195 (6)	0.0169 (6)	0.0136 (6)	0.0011 (6)	−0.0012 (6)	0.0028 (5)
C4	0.0232 (7)	0.0236 (7)	0.0173 (7)	−0.0037 (6)	0.0020 (6)	0.0017 (6)
C5	0.0144 (6)	0.0143 (6)	0.0160 (6)	0.0004 (6)	0.0003 (6)	−0.0005 (5)
C6	0.0117 (6)	0.0151 (6)	0.0146 (6)	−0.0003 (5)	0.0006 (6)	0.0005 (5)
C7	0.0099 (5)	0.0168 (6)	0.0139 (6)	0.0004 (5)	0.0020 (6)	0.0000 (5)
C8	0.0249 (8)	0.0185 (6)	0.0212 (7)	−0.0053 (6)	0.0008 (6)	0.0003 (5)
C9	0.0082 (6)	0.0170 (6)	0.0154 (6)	−0.0009 (5)	−0.0003 (6)	−0.0002 (5)
C10	0.0150 (6)	0.0153 (6)	0.0183 (6)	0.0006 (6)	−0.0001 (6)	0.0003 (5)
C11	0.0131 (6)	0.0156 (6)	0.0190 (6)	−0.0003 (5)	−0.0002 (6)	0.0036 (5)
C12	0.0096 (6)	0.0158 (6)	0.0154 (6)	−0.0008 (6)	−0.0005 (6)	0.0004 (5)
C13	0.0287 (8)	0.0208 (7)	0.0137 (6)	−0.0034 (6)	0.0004 (6)	0.0009 (5)
N1	0.0183 (5)	0.0138 (5)	0.0122 (5)	0.0015 (5)	0.0001 (5)	0.0010 (4)
N2	0.0186 (5)	0.0137 (5)	0.0176 (5)	0.0017 (5)	0.0003 (5)	−0.0018 (4)
N3	0.0240 (6)	0.0136 (5)	0.0137 (5)	−0.0014 (5)	0.0036 (5)	0.0002 (4)
N4	0.0135 (5)	0.0160 (5)	0.0164 (5)	0.0004 (5)	−0.0007 (5)	0.0017 (4)
N5	0.0136 (5)	0.0149 (5)	0.0141 (5)	−0.0005 (5)	0.0010 (5)	0.0013 (4)
O1	0.0363 (6)	0.0441 (7)	0.0152 (5)	−0.0122 (5)	0.0067 (5)	−0.0030 (5)
S1	0.02089 (16)	0.01483 (15)	0.01383 (15)	−0.00088 (14)	−0.00033 (14)	0.00007 (12)

### Geometric parameters (Å, °)

**Table:**

C1—O1	1.4369 (18)	C8—N3	1.4639 (17)
C1—C2	1.515 (2)	C8—H8A	0.9800
C1—H1A	0.9900	C8—H8B	0.9800
C1—H1B	0.9900	C8—H8C	0.9800
C2—C3	1.5373 (19)	C9—N5	1.3525 (17)
C2—H2A	0.9900	C9—C10	1.4066 (18)
C2—H2B	0.9900	C10—C11	1.3710 (18)
C3—N1	1.4652 (15)	C10—H10	0.9500
C3—C4	1.5484 (19)	C11—N4	1.3479 (17)
C3—H3	1.0000	C11—H11	0.9500
C4—O1	1.4262 (18)	C12—N4	1.3282 (16)
C4—H4A	0.9900	C12—N5	1.3375 (16)
C4—H4B	0.9900	C12—S1	1.7644 (13)
C5—N2	1.3162 (16)	C13—S1	1.7990 (13)
C5—C6	1.4119 (17)	C13—H13A	0.9800
C5—H5	0.9500	C13—H13B	0.9800
C6—C7	1.4080 (17)	C13—H13C	0.9800
C6—C9	1.4425 (17)	N1—N2	1.3811 (14)
C7—N1	1.3545 (16)	N3—H3N	0.858 (15)
C7—N3	1.3700 (16)		
			
O1—C1—C2	103.83 (12)	N3—C8—H8B	109.5
O1—C1—H1A	111.0	H8A—C8—H8B	109.5
C2—C1—H1A	111.0	N3—C8—H8C	109.5
O1—C1—H1B	111.0	H8A—C8—H8C	109.5
C2—C1—H1B	111.0	H8B—C8—H8C	109.5
H1A—C1—H1B	109.0	N5—C9—C10	120.10 (12)
C1—C2—C3	102.56 (12)	N5—C9—C6	117.54 (11)
C1—C2—H2A	111.3	C10—C9—C6	122.36 (12)
C3—C2—H2A	111.3	C11—C10—C9	117.16 (12)
C1—C2—H2B	111.3	C11—C10—H10	121.4
C3—C2—H2B	111.3	C9—C10—H10	121.4
H2A—C2—H2B	109.2	N4—C11—C10	123.96 (12)
N1—C3—C2	111.96 (11)	N4—C11—H11	118.0
N1—C3—C4	111.79 (11)	C10—C11—H11	118.0
C2—C3—C4	103.48 (10)	N4—C12—N5	128.31 (12)
N1—C3—H3	109.8	N4—C12—S1	118.95 (9)
C2—C3—H3	109.8	N5—C12—S1	112.74 (9)
C4—C3—H3	109.8	S1—C13—H13A	109.5
O1—C4—C3	106.78 (12)	S1—C13—H13B	109.5
O1—C4—H4A	110.4	H13A—C13—H13B	109.5
C3—C4—H4A	110.4	S1—C13—H13C	109.5
O1—C4—H4B	110.4	H13A—C13—H13C	109.5
C3—C4—H4B	110.4	H13B—C13—H13C	109.5
H4A—C4—H4B	108.6	C7—N1—N2	112.13 (10)
N2—C5—C6	113.04 (11)	C7—N1—C3	129.90 (11)
N2—C5—H5	123.5	N2—N1—C3	117.75 (10)
C6—C5—H5	123.5	C5—N2—N1	104.45 (10)
C7—C6—C5	103.86 (11)	C7—N3—C8	123.02 (12)
C7—C6—C9	127.08 (12)	C7—N3—H3N	109.4 (10)
C5—C6—C9	129.03 (12)	C8—N3—H3N	111.1 (11)
N1—C7—N3	125.53 (11)	C12—N4—C11	113.97 (11)
N1—C7—C6	106.50 (11)	C12—N5—C9	116.49 (11)
N3—C7—C6	127.87 (11)	C4—O1—C1	106.25 (11)
N3—C8—H8A	109.5	C12—S1—C13	101.21 (6)

### Hydrogen-bond geometry (Å, °)

**Table:**

*D*—H···*A*	*D*—H	H···*A*	*D*···*A*	*D*—H···*A*
N3—H3N···N5	0.858 (15)	2.157 (15)	2.8510 (14)	137.9 (14)
